# High-Dynamic-Range CT Reconstruction Based on Varying Tube-Voltage Imaging

**DOI:** 10.1371/journal.pone.0141789

**Published:** 2015-11-06

**Authors:** Ping Chen, Yan Han, Jinxiao Pan

**Affiliations:** 1 National Key Laboratory for Electronic Measurement Technology, North University of China, Taiyuan, 030051, China; 2 Key Laboratory of Instrumentation Science & Dynamic Measurement, North University of China, Taiyuan, 030051, China; Shanxi University, CHINA

## Abstract

For complicated structural components characterized by wide X-ray attenuation ranges, the conventional computed tomography (CT) imaging using a single tube-voltage at each rotation angle cannot obtain all structural information. This limitation results in a shortage of CT information, because the effective thickness of the components along the direction of X-ray penetration exceeds the limitation of the dynamic range of the X-ray imaging system. To address this problem, high-dynamic-range CT (HDR-CT) reconstruction is proposed. For this new method, the tube’s voltage is adjusted several times to match the corresponding effective thickness about the local information from an object. Then, HDR fusion and HDR-CT are applied to obtain the full reconstruction information. An accompanying experiment demonstrates that this new technology can extend the dynamic range of X-ray imaging systems and provide the complete internal structures of complicated structural components.

## Introduction

Computed tomography (CT) can be used in industrial nondestructive testing[1–2]. However, in the X-ray imaging system, under the limitation of dynamic range, the conventional single tube-voltage imaging method cannot capture all of the structure information for the complicated structural components. Because, for the complicated ones, the range of transmission thickness is wider, and single voltage is only corresponding to the certain attenuation thickness range. Namely, the ray energy of the fixed tube-voltage will not cover the wider thickness range about the complicated ones. That will be the contradiction between higher energy and lower density or thickness, or lower energy and higher density or thickness. Then the final image of the single tube-voltage will appear overexposed or underexposed[3][4]. Additionally, when the X-ray energy is higher, the initial gain correction coefficient will not be effective. Some “stripe-like” noise areas appear in the high-energy image. In other words, conventional fixed tube-voltage imaging will yield incomplete projections, and CT reconstruction based on such projections will be infeasible[5][6].

Currently, for these complicated ones, some compensating jackets or fillers are used, whose density is similar to the object, to make up the differences in thickness or density. Then, the entirety of the projection can be obtained at a fixed tube-voltage. However, these methods reduce the contrast because of the increased thickness[7][8]. In addition, for the sufficiently complicated objects, it is not easy to make the suitable compensating jackets and fillers.

Thus, some researchers use fusion algorithm to extend the dynamic range of detector based on multi-exposure image sequences with the different ray energy. These multi-exposure sequence are fused to the new HDR projection[9]. Philipp *et al*. have proposed a method of fusing variable-current X-ray images to extend the dynamic range[10]. Sisniega *et al*, combine two projection datasets with different current by a maximum likelihood estimation based on previous knowledge of the detector response about incoming radiation[11]. However, X-ray penetration primarily depends on the X-ray tube voltage, and varying the current is insufficient for the wider ray attenuation ranges. In addition, the presented method is dual-exposure imaging, which is based only on the manipulation of the volumetric data and combining these data via averaging, and doesn’t consider the phenomena of overexposure and underexposure. Moreover, to date, no practical application of this method has been used for the imaging of complicated structural components.

In this paper, we fuse the projection sequences with different voltage at the every projection angle to get the high-dynamic-range (HDR) projection, because one tube voltage is corresponding to the certain attenuation thickness, which is showed in the local effective area of projection. This HDR imaging method is similar to the visible-light imaging with multiple exposures [12].

## Imaging Based on Multi Tube-Voltage

### Imaging principle and system

**Fig 1:**
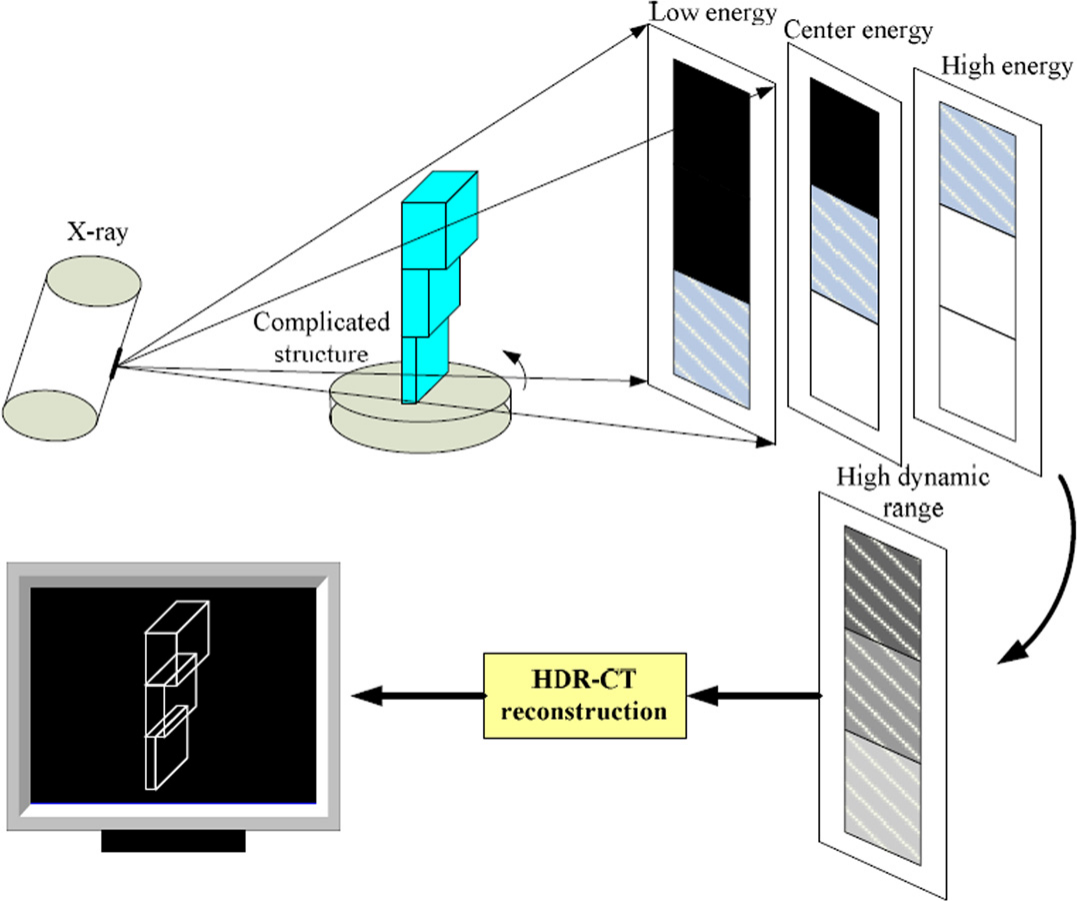
The principle of HDR-CT imaging. For the complicated components, get multi-voltage sequences at one projection angle, which are responding to different thickness. Then by HDR fusion and CT reconstruction, get the full information.

In X-ray imaging, if the tube voltage is higher, the penetration depth will be greater, and the density suitable for imaging will be higher. So, for complicated ones, we only get some local effective projection information, where the partial thickness or density can normally imaging, corresponding to the fixed voltage. So we can design the varying tube-voltage imaging method to get all local area, which is showed in Fig 1.

In Fig 1, under the limitation of dynamic range of imaging system, the different thickness needs the different ray energy to match. Then the full information can be obtained by HDR-fusion and HDR-CT. So adjust the tube voltage from low to high voltage at every rotation angle using a conventional X-ray CT scanning. In turn, all the local projections are showed in the multi-voltage sequences. However, to ensure the continuity of the adjacent energy images, the tube voltage must be gradually increased with a small voltage interval.

### Multi tube-voltage projection acquisition

**Fig 2:**
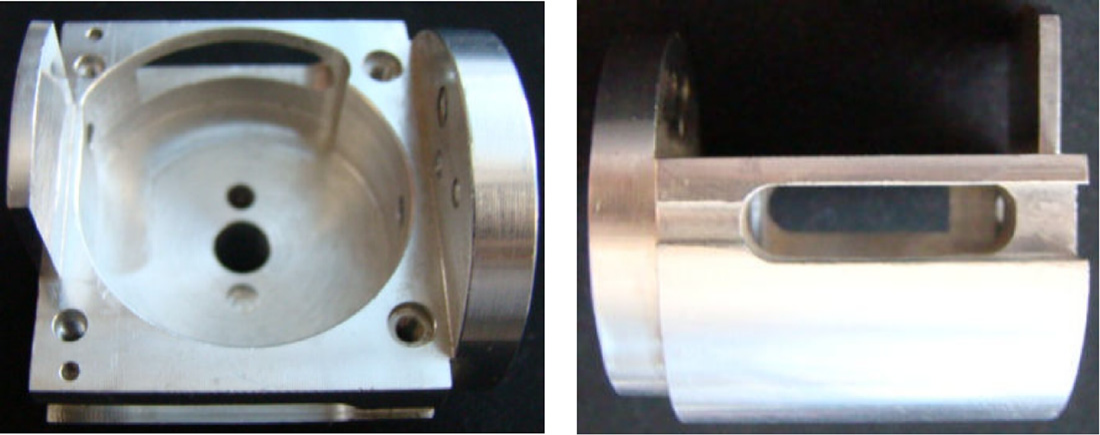
The complicated sample. The thickness is the notable difference. The bottom is solid, the center is hollowed-out, and the top is irregular.

**Fig 3:**
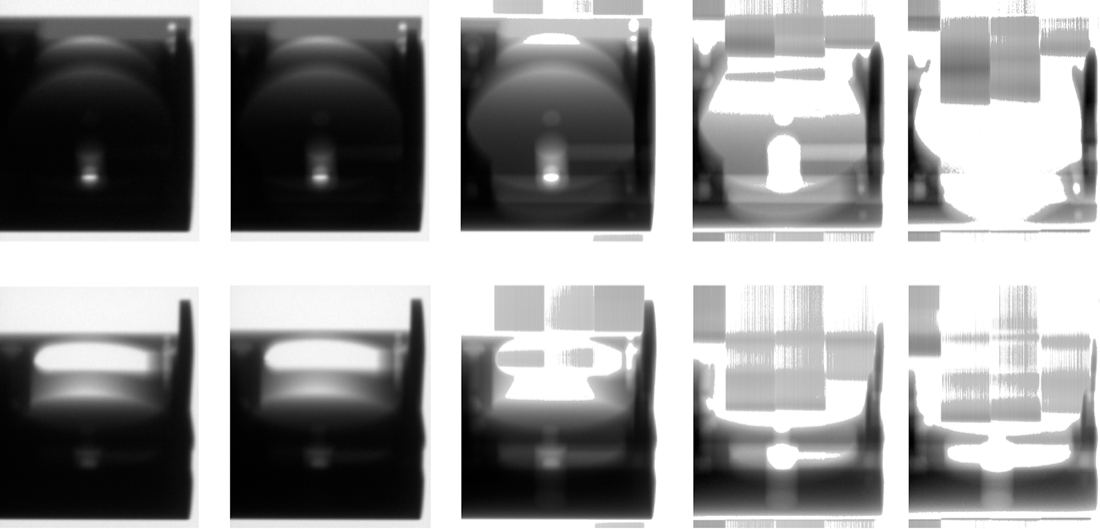
The image sequences collected at varying energies. Each row corresponds to a different projection angle (0° and 90°), and each column corresponds to a different transillumination voltage (60 kV, 70 kV, 80 kV, 90 kV, and 100 kV).

**Fig 4:**
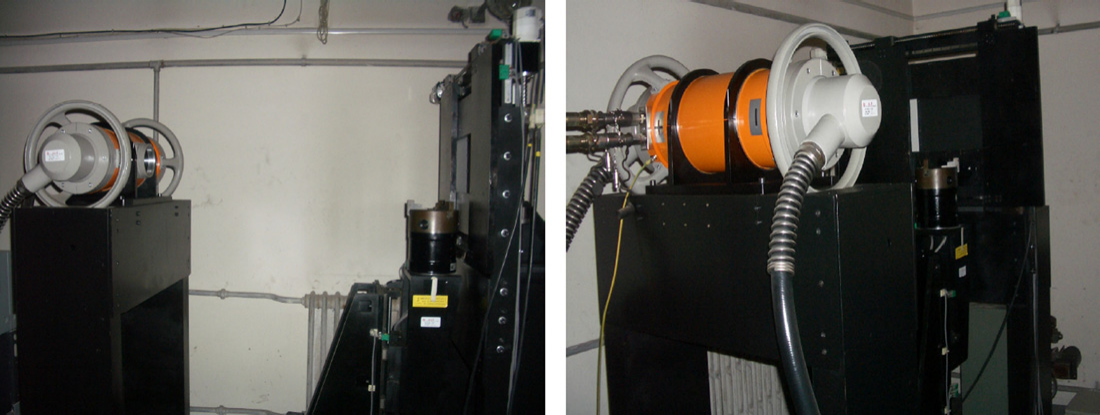
The experiment CT system. This system is composed by X-ray (GE ISOVOLT 450KV) and detector (VARIAN PaxScan2520).

Based on the above varying tube-voltage imaging, for the workpiece depicted in Fig 2, image sequences of cone-beam scanning are captured from 60 kV to 100 kV in step of 10 kV at every projection angle (total 360°, interval 1°), which are shown in Fig 3. These images are acquired by a 12-bit imaging system in our laboratory (Fig 4).

**Fig 5:**
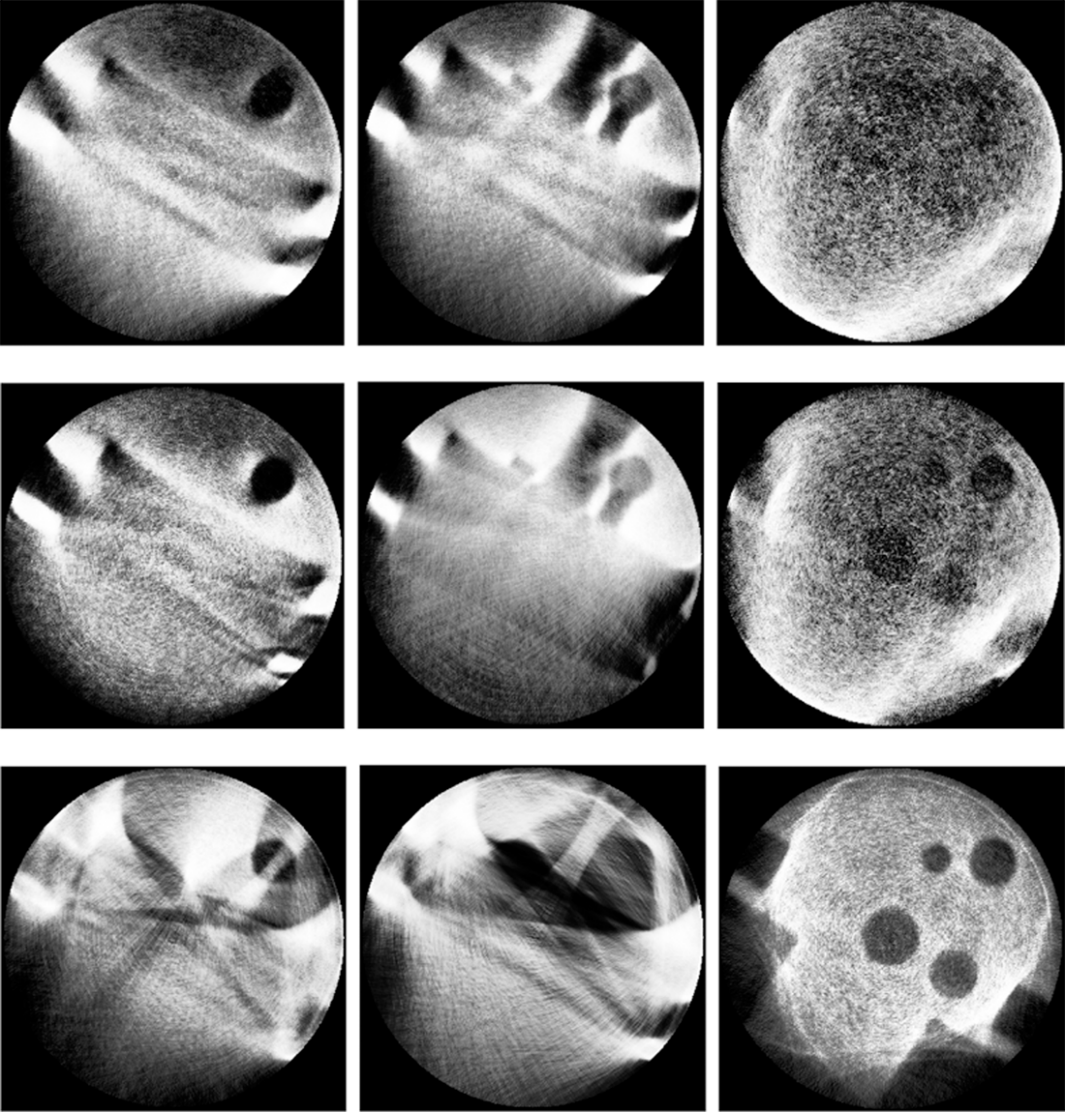
Single-voltage CT images with different energy and different part. Every column is the 60kV, 80kV and 100kV at the same prat. Every row is the bottom, center, and top part at the same voltage.

As seen in Fig 4, because of the wider variations of the effective thickness, the single voltage can’t make ray energy match with all thickness, and the image sequences exhibit local effectivity. Such as, these projections contain part construct information, under-exposed area (back area), overexposed area (white area) and “strip-like” noise (strip area). Namely, any projection is not integrated. By these projections, the CT reconstruction results are intolerable in Fig 5.

From Fig 5, in the CT reconstruction result with low voltage, the penetrating power is so low that the innermost structure of the sample cannot be reconstructed. Moreover for higher voltages, only the inner information is obtained, the outer edge cannot be successfully reconstructed. So the convention CT method is invalid.

## HDR Fusion

### Gray gain figure between sequences

**Fig 6:**
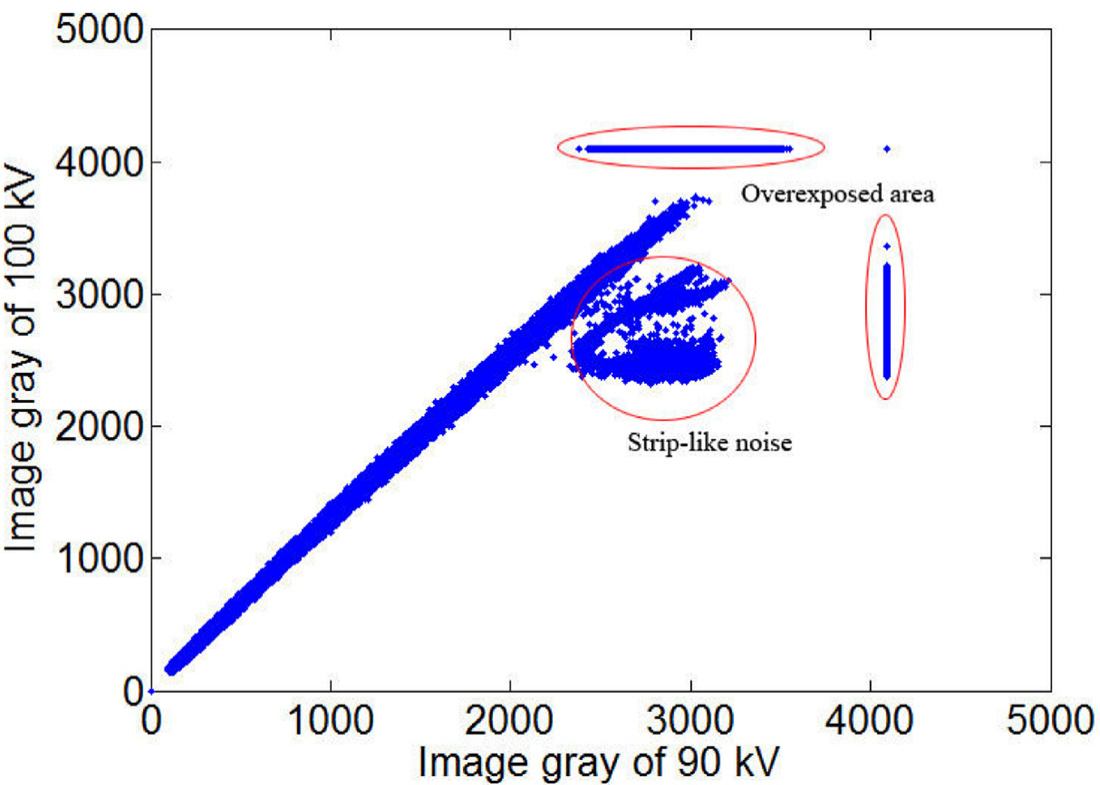
The corresponding gray figure between 90kV and 100 kV at 0°. Horizontal axis represents the gray of one tube-voltage image, and vertical axis represents the next tube-voltage. These points represent the gray of one point about object in the adjacent two frames images.

Because the increasing interval of tube-voltage is smaller, there should be same effective area between two adjacent frames. The other area will be showed as overexposed, underexposed or tripe noise in the one of adjacent frames. Also this same effective area exists in gray gain because of the increasing of tube-voltage. However the gray should be same at this same area at one tube-voltage. So in order to show this gray gain, we interpolate point by point to obtain the corresponding gray figure between any two adjacent frames, as shown in Fig 6.

From the corresponding projection of Fig 6 (in Fig 4), because of the higher ray energy, the image area of the thinner thickness will appear overexposed and strip-like noise. Specially, the transverse point set is the overexposed area in 100kV, which is the normal area in 90kV. Also there are some vertical points and “strip-like” noise points, which are the overexposed area in 90kV. The approximate linear point set represents the local effective area.

**Fig 7:**
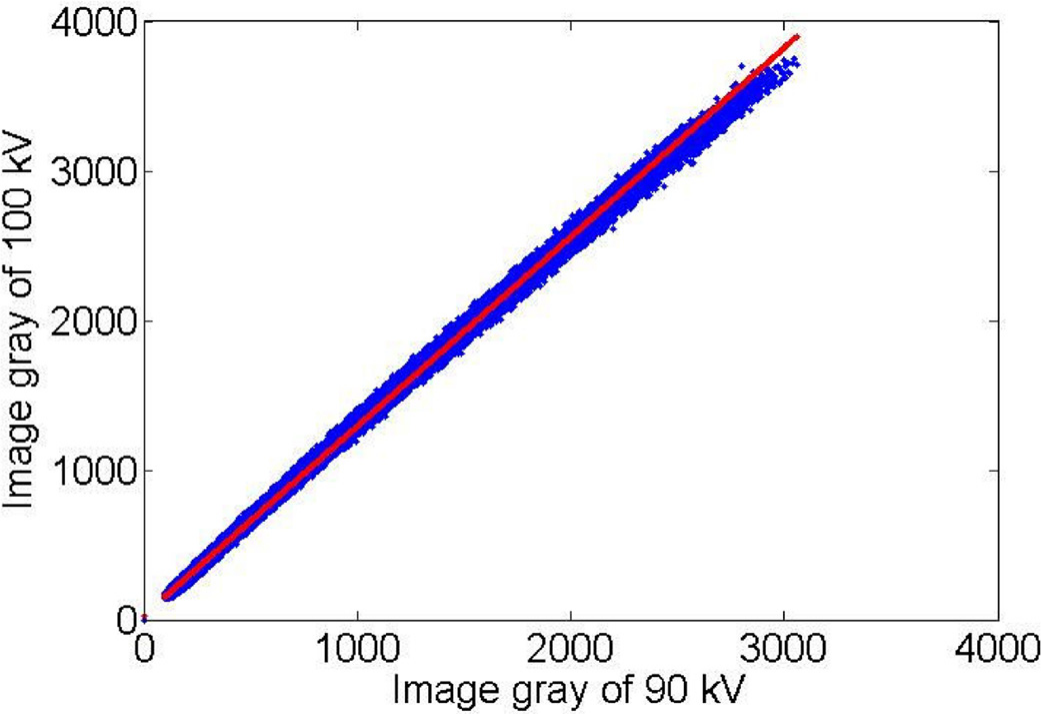
The processed gray figure about 90kV and 100kV. That has excluded the non-interesting area (overexposed area and “strip-like” noise).

From Fig 6, we should calculate the gray gain coefficient from the approximate linear point set. Here we can use the linear fit to obtain the gray gain coefficient. However, the gray values of the overexposed regions and the “strip-like” noise (labeled in Fig 6) will affect the fitting precision. Therefore, we must eliminate these factors. Also from Fig 4, the overexposed and noise area is the effective region in lower-voltage image. Therefore, we can define the overexposed area, whose gray is more than 4000. And this overexposed region is the effective area at lower voltages. So in the image of the lower voltage, define the overexposed area by gray threshold. Then use this defined region to eliminate the corresponding areas in the higher-voltage image. The processed gray of 90kV and 100kV figure is presented in Fig 7.

### Image sequences fusion based on gray gain figure

Using the data in Fig 7, we defined the processed point set is *F*_*i*_ (*i =* 1,2,*…*,*n*). The final fusion HDR projection *F* can be defined.

$$F=\omega_{1}F_{1}+\omega_{2}F_{2}+\cdots +\omega_{n}F_{n}$$ (1)

Where, *ω*_*i*_ is the weighting factor. If no constraint of dynamic range, in order to ensure all construct normal imaging, the imaging tube-voltage should be same with *F*_*n*_. Then the thickness is thinner, the image gray is higher. So in Eq (1), *ω*_*n*_ = 1, sequence *F*_1_, *F*_2_, *…*, *F*_n-1_ respectively add to *F*_*n*_ with the corresponding coefficient *ω*_*i*_. And the relationship of *ω*_*i*_ is written as Eq (2).

$$\omega_{1}>\omega_{2}>\cdots >\omega_{n}=1$$ (2)

Also, from linear fitting (Fig 7), we can obtain the gray-gain coefficients *a*_*i*_ and the gray offsets *b*_*i*_ about *F*_*i*_ and *F*_i+1_.

$$F_{i+1}=a_{i}F_{i}+b_{i}.$$ (3)

Eq (3) is represent the gray of *i* ^*st*^ tube-voltage at (*i+*1)^*st*^ tube-voltage. By Eq (3), we can calculate the fused gray *ω*_*i*_ *F*_*i*_. For example, for the last two frames *F*_n-1_ and *F*_*n*_, *F*_n-1_ should add to *F*_*n*_ by gray gain. The fusion image *F’* is:

$$F^{'}=\left(F_{n-1}a_{n-1}+b_{n-1}\right)+F_{n}$$ (4)

As so, all processed sequences are added to *F*_*n*_, and the full image *F* can be expressed as:

$$\begin{array}{l} F=\left(F_{1}\prod_{i=1}^{n-1}a_{i}+b_{1}\prod_{i=2}^{n-1}a_{i}+b_{n-1}\right)+\left(F_{2}\prod_{i=2}^{n-1}a_{i}+b_{2}\prod_{i=3}^{n-1}a_{i}+b_{n-1}\right)+\cdots +\left(F_{n-1}a_{n-1}+b_{n-1}\right)+F_{n}\\ \;=\left(F_{1}\prod_{i=1}^{n-1}a_{i}+F_{2}\prod_{i=2}^{n-1}a_{i}+\cdots +F_{n-1}a_{n-1}+F_{n}\right)+\left(b_{1}\prod_{i=2}^{n-1}a_{i}+b_{2}\prod_{i=3}^{n-1}a_{i}+\cdots +(n-1)b_{n-1}\right) \end{array}.$$ (5)

**Fig 8:**
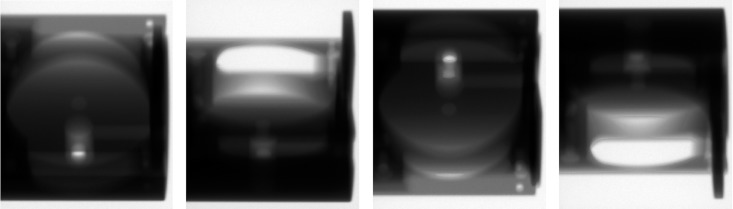
The fused HDR projections at 0°, 90°, 180°, and 270°.

Then we can obtain the full HDR-CT projection at every angle, as shown in Fig 8.

## HDR-CT

### Directly reconstruction

**Fig 9:**
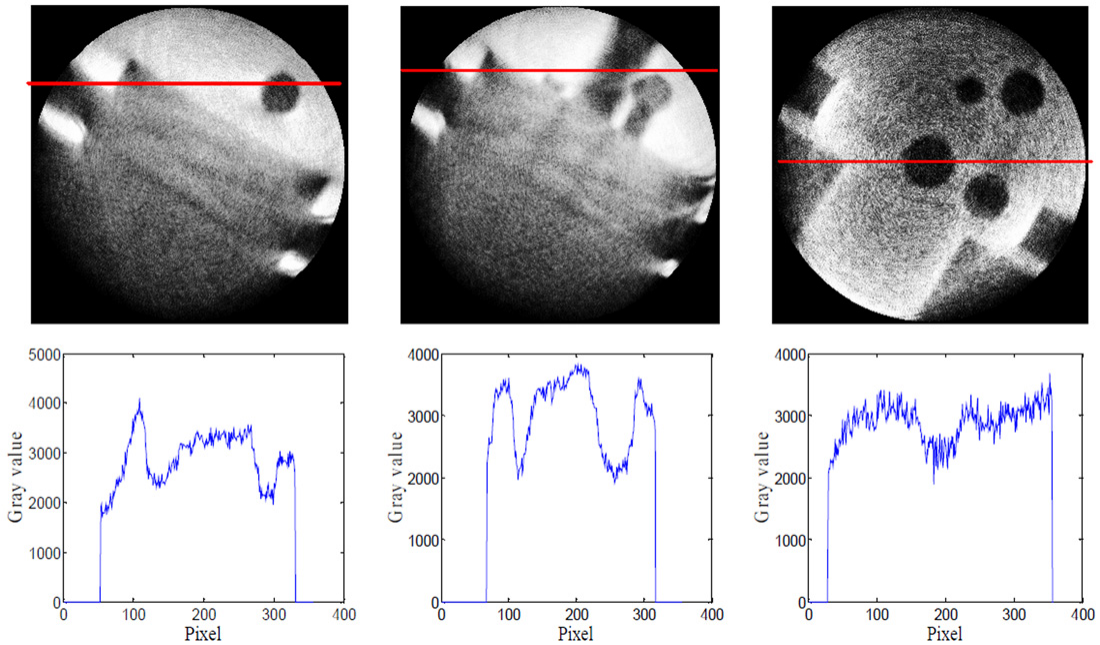
HDR-CT reconstruction results. The first row CT image from left to right is corresponding to bottom, center and top about workpiece, The second row is respectively the gray curve at the indicated line. In these CT image the construction is full, but the image quality is poor, and noise level is higher.

Through HDR fusion, we can obtain CT projections with high integrity of information. Therefore, we can then apply a conventional reconstruction algorithm, such as FBP, FDK, ART [13–15]. Here, for the HDR-projection (Fig 8), using FDK reconstruct. The result is presented in Fig 9.

Unlike the results present in Fig 5, the workpiece is perfectly reconstructed in these images. However, the edges are not clear, and the noise level is higher. These issues of image quality arise because in HDR fusion, the lower-energy images are combined with the highest-energy images using coefficients greater than 1, as Eq (2). This causes the noise level in the HDR image to increase. Therefore, to avoid noise amplification, we should improve the method used to weight the coefficients.

### HDR-CT with logarithm transformation

According to Beer’s law, a negative logarithm conversion can be applied to compress a dynamic range. Also, in CT reconstruction, generally the acquired projection is carried out logarithm transformation by background gray. So we can carry out logarithm for processed sequences *F*_*i*_. so

$$p_{i}=\ln\frac{I_{i0}}{F_{i}},$$ (6)

Where *I*_*i*0_ is the background gray level at the *i* ^th^ voltage. As Eq (6), the noise level of every frame will decrease. And *p*_*i*_ is a decreasing function about *F*_*i*_. So in Eq (2), we can get the new fusion weighting coefficients *ν*_*i*_.

$$p=\nu_{1}p_{1}+\nu_{2}p_{2}+\cdots +\nu_{n}p_{n}$$ (7)

The relationship is rewritten as:

$$\nu_{1}>\nu_{2}>\cdots >\nu_{n}=1$$ (8)

**Fig 10:**
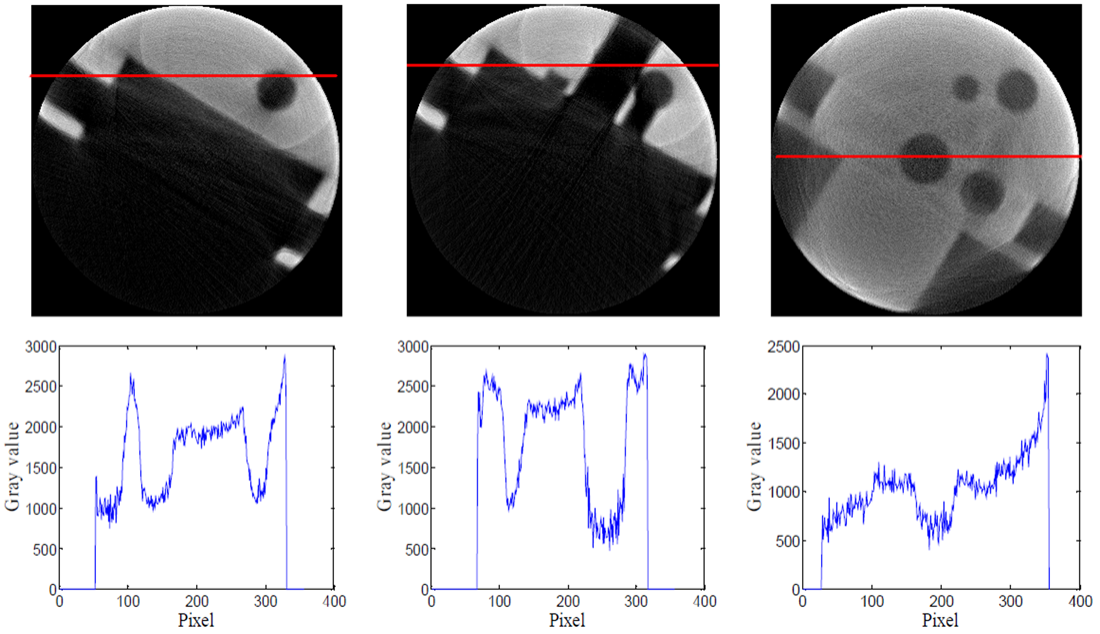
CT images obtained using the improved fused images. The first row CT image from left to right is corresponding to bottom, center and top about workpiece, The second row is respectively the gray curve at the indicated line. Relative to Fig 8, image quality and noise have major improvement

The subsequent line fitting and HDR-fusion is based on the logarithm result *p*_*i*_. By Eq (8), the minimal energy image multiplies the minimal coefficient, and all coefficients are less than 1. So we can control the noise amplification. To demonstrate that such images yield improved reconstruction quality, we applied the FDK algorithm to reconstruct the improved images. The results are presented in Fig 10.

**Fig 11:**
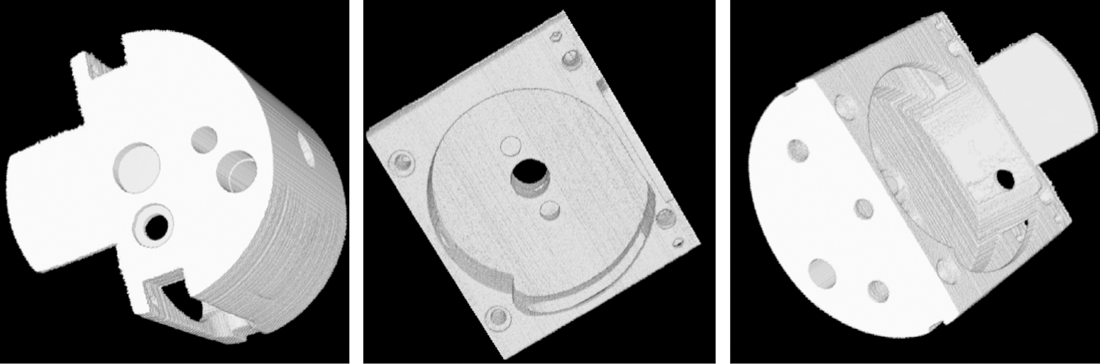
3D visualizations of the real workpiece at various viewing angles.

**Fig 12:**
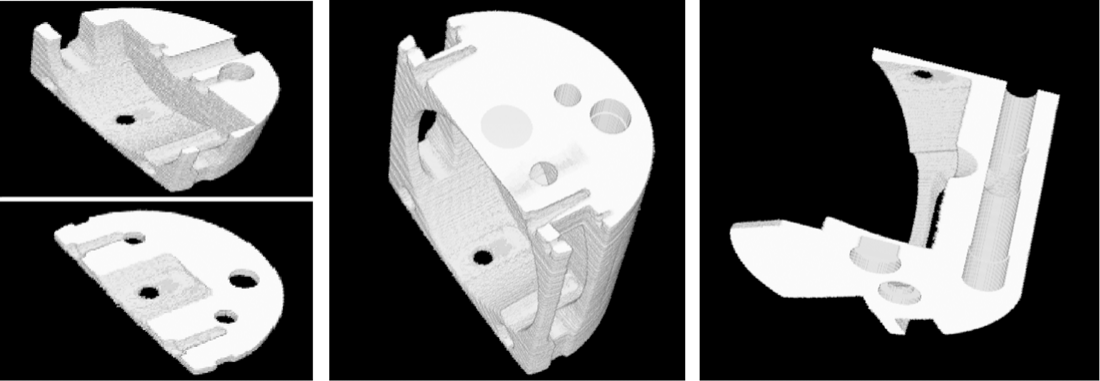
3D sections viewed from various axes.

From Fig 10, the object’s details are notably clearer, and the contrast of CT images is higher. So we can use threshold segmentation and VTK to obtain 3D visualizations, which is shown in Fig 11 and Fig 12. From the 3D visualization (Figs 11 and 12), we can get the full construct information about the complicated workpiece.

## Conclusion

For an object that spans a broad range of X-ray attenuation, conventional fixed tube-voltage CT imaging cannot obtain all of its structural information, because of the mismatch among the X-ray energy, thickness and dynamic range of the imaging system. Therefore, underexposed and overexposed regions will appear. That will affect the CT reconstruction. But by varying tube-voltage imaging, we can get all local information about object, whose attenuation thickness is match with the ray energy from the multiple different voltage imaging. So for the paper’s sample, at each projection angle, we collected seven images with X-ray energies ranging from 60 kV to 100 kV with 10kV interval.

In order to solve gray consistency at different voltage, the gray gain figure is proposed. We removed any invalid areas based on the linear character of the gray gain figure. Then, based on the knowledge that the same area should appear at the same gray level at a given voltage, we computed the ratio coefficients to determine the gray mapping relations between adjacent voltages to obtain HDR projection.

Further, to decrease the noise of HDR projection, using negative logarithm conversion to modify the fusion coefficients to reduce the noise level. And by FDK algorithm to reconstruct CT image, whose noise and contrast of CT images obtained using negative logarithm conversion are improved with respect to direct reconstruction. Using the images obtained in this manner, we could further extract the structural information and obtain a 3D visualization. The experiment shows this new approach is suitable for the imaging of complicated object with the wider varying thickness, which cannot be fully imaged using conventional single tube-voltage CT. Our method achieves far superior results without requiring any hardware upgrade.

However, because of multi-spectrum at every tube-voltage, our reconstruction results exists beam hardening artifact. So at present we only realize qualitative detection for complicated structure component. Next we will research multi-energy reconstruction algorithm based on varying tube-voltage to improve CT quality, which can meet size measurement and quantitative determination.
